# A Method for Trust-Based Collaborative Smart Device Selection and Resource Allocation in the Financial Internet of Things

**DOI:** 10.3390/s25134082

**Published:** 2025-06-30

**Authors:** Bo Wang, Jiesheng Wang, Mingchu Li

**Affiliations:** 1 School of Applied Technology, University of Science and Technology Liaoning, Anshan 114051, China; 2 Julong Co., Ltd., Anshan 114051, China; 3 School of Computer Science and Technology, Dalian University of Technology, Dalian 116024, China; 4 School of Electronic and Information Engineering, University of Science and Technology Liaoning, Anshan 114051, China

**Keywords:** Financial Internet of Things, edge computing, trust evaluation, resource allocation, coalition game

## Abstract

With the rapid development of the Financial Internet of Things (FIoT), many intelligent devices have been deployed in various business scenarios. Due to the unique characteristics of these devices, they are highly vulnerable to malicious attacks, posing significant threats to the system’s stability and security. Moreover, the limited resources available in the FIoT, combined with the extensive deployment of AI algorithms, can significantly reduce overall system availability. To address the challenge of resisting malicious behaviors and attacks in the FIoT, this paper proposes a trust-based collaborative smart device selection algorithm that integrates both subjective and objective trust mechanisms with dynamic blacklists and whitelists, leveraging domain knowledge and game theory. It is essential to evaluate real-time dynamic trust levels during system execution to accurately assess device trustworthiness. A dynamic blacklist and whitelist transformation mechanism is also proposed to capture the evolving behavior of collaborative service devices and update the lists accordingly. The proposed algorithm enhances the anti-attack capabilities of smart devices in the FIoT by combining adaptive trust evaluation with blacklist and whitelist strategies. It maintains a high task success rate in both single and complex attack scenarios. Furthermore, to address the challenge of resource allocation for trusted smart devices under constrained edge resources, a coalition game-based algorithm is proposed that considers both device activity and trust levels. Experimental results demonstrate that the proposed method significantly improves task success rates and resource allocation performance compared to existing approaches.

## 1. Introduction

The Financial Internet of Things (FIoT) has emerged as a transformative paradigm in modern finance, enabling intelligent sensing, real-time decision-making, and automated service delivery through interconnected smart devices. Each smart device offloads tasks to cooperating devices with idle resources via end–edge or end-to-end collaboration mechanisms, aiming to reduce service latency caused by resource constraints. However, as device deployment scales up and becomes more complex, ensuring system reliability, data security, and efficient resource management has become increasingly challenging. In addition, some cooperative service devices may exhibit malicious behaviors, such as disrupting networks or degrading services, thereby compromising system performance. Some devices may attempt to gain substantial profit by offering deceptive or misleading services. Some devices may intermittently offer legitimate, deceptive, or malicious services. Therefore, a robust scheme capable of resisting such risks and attacks in FIoT environments is urgently needed. To address the challenge of resisting malicious behaviors and attacks in the FIoT, this paper proposes a trusted smart device selection algorithm that integrates both subjective and objective trust mechanisms with a dynamic blacklist and whitelist, leveraging domain knowledge and game theory. Given the asymmetry in trust values, the trust model considers subjective and objective factors, including service quality, tolerance, recognition, and temporal dynamics, to enhance trust evaluation accuracy. In addition, a dynamic blacklist and whitelist transformation mechanism is introduced to capture behavioral changes in collaborative devices and update trust records accordingly.

With the widespread application of artificial intelligence (AI) technologies, numerous AI algorithms, such as facial recognition, currency classification, and financial risk assessment, are frequently employed in the FIoT. Although existing methods address the resource allocation problem for certain trusted cooperative devices, significant challenges remain in large-scale FIoT environments. Due to the constrained resources of trusted edge devices operating under collaborative mechanisms, efficient resource allocation among trusted smart devices becomes critical. To tackle this issue, this paper proposes a coalition game-based resource allocation algorithm that considers device activity levels and trust values to optimize the allocation of limited collaborative edge resources.

In summary, this paper investigates trusted smart device selection and efficient resource allocation for collaborative devices in the FIoT under a collaboration mechanism. We emphasize that the integration of these components in our algorithm is specifically designed to address challenges in the FIoT, particularly in dealing with dynamic malicious behaviors and resource allocation in edge devices. Previous works have often treated trust models and resource allocation separately or used them in a static context, without considering dynamic blacklist/whitelist adaptations or the integration of coalition game theory for real-time resource allocation. This approach enhances the security and reliability of smart devices in FIoT, contributing to overall system stability within collaborative environments.

The main contributions of this paper are as follows:

- To ensure secure and reliable collaboration among intelligent devices in the FIoT, a comprehensive trust model is proposed. This model quantifies trust based on three distinct but complementary components: direct trust, indirect trust, and aggregate trust. Direct trust is calculated based on the historical interactions and behavioral patterns observed between devices, reflecting firsthand experience. Indirect trust is derived from recommendations or feedback provided by other nodes within the network, allowing trust to be estimated even in the absence of direct interactions. Aggregate trust serves as a unified measure, synthesizing direct and indirect trust to produce a holistic evaluation of a device’s trustworthiness. This layered approach not only improves the accuracy of trust assessments but also enhances the system’s resilience to malicious behavior, misinformation, and reputation manipulation. A trusted device selection algorithm that integrates both trust and black- and whitelist mechanisms is introduced. The proposed algorithm enhances the system’s capability to withstand attacks.
- Building upon the proposed trust model, a trusted device selection algorithm is developed to identify suitable collaboration partners among FIoT devices. This algorithm integrates both the computed trust values and a black- and whitelist mechanism, offering a dual-layered security framework. The whitelist includes devices with a verified history of trustworthy behavior, ensuring priority in resource allocation and task assignment. The blacklist consists of devices that have been identified as compromised, untrustworthy, or malicious, thereby preventing them from participating in collaborative processes. This hybrid mechanism enables dynamic and adaptive device selection, significantly strengthening the system’s ability to defend against a wide range of attacks. The result is a robust and scalable method for maintaining the integrity and reliability of device interactions in FIoT environments. To address the dynamic nature of device behavior in FIoT environments, this paper also proposes a dynamic black- and whitelist transformation mechanism. Unlike static approaches, this mechanism enables the real-time adaptation of trust boundaries by continuously monitoring device behavior and updating list memberships accordingly. This dynamic transformation mechanism ensures that the trust system remains both flexible and responsive to evolving threats and behavioral shifts. It mitigates the risks associated with fixed classifications and provides a robust defense against strategic attackers who may alternate between benign and malicious behavior to evade detection.
- To optimize the utilization of limited resources among a dynamically changing set of trusted devices, a resource allocation algorithm based on coalition game theory is proposed. In this framework, devices form coalitions based on mutual trust and resource needs, cooperating to share resources in a way that maximizes the overall utility of the group. This approach allows for flexible, dynamic, and decentralized resource management, which is critical in heterogeneous and large-scale FIoT networks. By leveraging coalition formation strategies, the system can adapt to changing demands and trust landscapes in real-time, ensuring sustained performance and security.

The rest of this paper is organized as follows. Section 2 reviews and examines the related works. In Section 3, we present our proposed methods. Section 4 presents the results of our analysis, simulation, and experimental example. Finally, Section 5 provides conclusions.

## 2. Related Work

Trust management in edge computing and the Internet of Things (IoT) has been extensively explored to address the challenges of security, reliability, and collaboration among distributed devices. Yang et al. [1] proposed a task offloading scheme based on active trust detection, where trust is dynamically calculated based on the outcomes of previous interactions. The task offloading strategy based on trust detection proposed in this article greatly improves the success rate of tasks. Sun et al. [2] developed an access control model that integrates mutual trust between nodes and users within edge computing environments. By combining trust management with a role-based access control (RBAC) model, they addressed the multi-domain nature of edge computing, implementing both intra-domain and cross-domain access control policies based on trust, thus extending and improving traditional RBAC. Li et al. [3] introduced the use of blockchain technology to store, disseminate, and update trust information in a decentralized manner, enhancing the evaluation of edge servers’ trustworthiness. Their trust model incorporates factors such as honesty, behavior, and reliability. Bounaira et al. [4] proposed an edge caching system enhanced by trust management using blockchain, aiming to secure the caching process through the establishment of bidirectional trust between content providers and edge servers. Their trust management system is based on a direct trust score calculated using the Subjective Three-Valued Logic Scheme (3VSL), which enhances confidence among caching participants.

Resource allocation in edge–cloud collaboration IoT environments has been widely studied due to the increasing demand for low latency, high efficiency, and system adaptability. A variety of optimization, artificial intelligence, and trust-based methods have been proposed in the literature. Geng et al. [5] investigated a dynamic resource allocation scheme involving multiple tasks. Under the long-term constraints of energy consumption and system cost, a cloud-edge collaborative offloading optimization problem was put forward to minimize the average task delay. The work put forward by Mageswari et al. [6] is centered on the issue of IoT resource allocation and scheduling and aimed to minimize the total communication cost between the gateway and the resource through the elephant herding optimization algorithm, thereby achieving the optimal resource allocation in IoT. Mostafa et al. [7] employed a reinforcement learning algorithm, allowing it to adapt to the inherent demand fluctuations and operating condition characteristics of IoT systems. A custom-designed reinforcement learning model was integrated within the framework, enabling it to acquire the optimal allocation policy through continuous interaction with the environment. This facilitated efficient load balancing and minimized latency without human intervention. Elgendy et al. [8] proposed a new framework for offloading intensive computing tasks from mobile devices to the cloud. The framework uses an optimization model to dynamically determine offloading decisions based on four main parameters: energy consumption, CPU utilization, execution time, and memory usage. In addition, a new security layer is provided to protect the data transmitted in the cloud from any attacks. Kang et al. [9] used consortium block-chain and smart contract technology to achieve secure data storage and sharing in vehicle edge networks. These technologies effectively prevent unauthorized data sharing. Liu et al. [10] addressed the challenges of task scheduling and resource allocation in ultra-dense edge–cloud networks, which encompass micro (macro) base stations and various device users under 5G technology. They proposed a two-level scheduling framework to accommodate the dynamic nature of user tasks. Zhou et al. [11] primarily studied the resource allocation optimization in a complex Internet of Things environment, addressing the challenge of task offloading and resource allocation for hybrid energy collaborative tasks assisted by drones and multiple clouds. They proposed a collaborative task offloading and resource allocation algorithm leveraging artificial intelligence technology, which jointly determines task offloading and energy acquisition. Deng et al. [12] proposed a reinforcement learning approach for dynamic resource allocation at the edge of trusted IoT systems, aiming to enhance the reliability and efficiency of resource allocation. Liu et al. [13] studied resource allocation in edge computing within the IoT network through machine learning, focusing on enhancing the efficiency of resource allocation using machine learning techniques, and achieved promising results. Xu et al. [14] proposed a resource allocation algorithm that achieves cost-efficient private cloud resource allocation and cost-efficient public cloud resource allocation in a collaborative cloud-edge environment, utilizing the deep reinforcement learning algorithms of deep deterministic policy gradient and P-DQN.

Furthermore, additional security threats targeting FIoT systems can be identified across the following dimensions: First, significant challenges in ensuring the security of FIoT firmware arise from its closed-source nature, limited device accessibility, and hardware heterogeneity. Financial infrastructure devices, such as point-of-sale (POS) terminals and mobile payment nodes, heavily rely on firmware integrity; any compromise may lead to transaction manipulation, the injection of fraudulent data, or the deployment of persistent malware [15]. Second, the integration of artificial intelligence (AI) large language models (LLMs) into FIoT environments is increasingly prevalent. The security of AI-driven agents is important. As these agents are progressively adopted in FIoT applications—such as fraud detection systems and automated pricing mechanisms—their behavioral complexity introduces new challenges concerning trustworthiness and regulatory compliance. Malfunctioning or malicious behaviors may result in legal violations or systemic financial instability [16]. Third, LLMs such as ChatGPT present considerable risks when deployed within critical infrastructures. When integrated with FIoT systems, particularly those incorporating conversational AI interfaces, vulnerabilities may undermine user confidentiality, compromise transactional reliability, and impair the accuracy of financial decision-making [17].

Despite these advancements, most existing schemes primarily focus on either trust evaluation or resource coordination in isolation. They often lack the ability to adapt to dynamic malicious behavior or efficiently handle resource constraints in large-scale FIoT environments. To address these challenges, this paper proposes a trusted collaborative smart device selection algorithm that integrates both subjective and objective trust mechanisms with a dynamic black- and whitelist system. Furthermore, by considering device activity levels and trust values, a coalition game-based resource allocation algorithm is introduced to optimize the allocation of limited edge resources. This approach not only improves resistance to malicious attacks but also enhances task success rates and resource utilization, particularly in resource-constrained edge settings.

## 3. Proposed Methods

### 3.1. System Scenario

**Figure 1:**
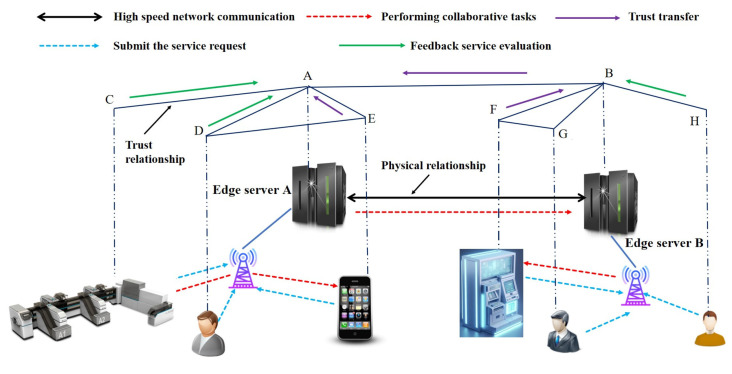
Financial IoT collaborative edge computing scenario.

In the trust-based collaborative edge computing scenario of the FIoT, the architecture comprises three primary components: end users, edge servers, and collaborative service devices. As illustrated in Figure 1, end users access edge servers wirelessly to request computational or storage services. Upon completion of the submitted tasks, end users provide service feedback to the edge servers. Before initiating a new task, end users send a request to the edge server to query the credibility of relevant collaborative service devices. The edge server continuously monitors the behavioral performance of these devices, collects evaluation feedback from various sources, and aggregates the data to calculate a trust value for each device. Based on these trust evaluations, untrusted or malicious devices are filtered out, and resources from the selected collaborative service devices are allocated accordingly. Our target scenario is collaborative service and data sharing among financial IoT devices (e.g., ATMs, POS, trading terminals, etc.). We specifically focus on the FIoT because of its unique characteristics, such as the high demand for security, trust, and efficient resource allocation due to the sensitive nature of financial transactions. The model applies to broader IoT but is tuned to financial scenarios where trust and reliability are critical.

FIoT edge computing scenarios often involve various types of users. For generalization, we consider two types of users in the system: ordinary users and malicious users. Ordinary users deliver effective services and provide accurate evaluation feedback. In contrast, malicious users offer ineffective services and misleading reviews. This paper examines four malicious attack models.

Bad-mouthing attacks [18]: In this model, independent malicious users provide invalid services and negative trust ratings for legitimate devices during interactions. This reduces the likelihood of good devices being chosen as service providers.

Good-mouthing attacks [19]: In this model, independent malicious users deliver invalid services to one another while assigning positive trust ratings. This behavior increases the likelihood of malicious devices being selected as service providers.

Ballot-stuffing attacks [20]: In this model, colluding malicious devices exchange favorable reviews. When chosen as service providers, they deliver ineffective services.

Selective behavior attacks: In this model, a malicious device may offer a valid service to enhance its trust value, increasing the likelihood of subsequently providing invalid services.

The choice of attack models such as bad-mouthing, good-mouthing, ballot-stuffing, and selective behavior attacks was based on their relevance to the manipulation of trust scores in collaborative edge computing environments. These attacks are representative of the behaviors that malicious devices or users could exhibit in the FIoT, targeting the trust and reputation management systems. While other attacks, such as Sybil and DoS attacks, could be explored in future work, they were not considered in this study due to their increased complexity and specific assumptions regarding network structures.

### 3.2. Trust Mechanism Design

#### 3.2.1. Subjective Direct Trust

1. Direct Trust Computation

After completing a task, requester i will evaluate the collaborative service device j based on its service quality and assign a score. The set of evaluations $\Theta =\left\{f\left|f\in \left[-2,2\right]\right.,f\in Ζ\right\}$ is defined as the set of evaluation results. The evaluation results $tr\left(i,j\right)$ belong to the set Θ. A multi-dimensional evaluation mechanism is represented by Equation (1).

(1) $$tr\left(i,j\right)=\left\{\begin{array}{cc} 2 & great\;satisfaction\\ 1 & satisfaction\\ 0 & general\;case\\ -1 & dissatisfaction\\ -2 & Be\;very\;dissatisfied \end{array}\right.$$

The aggregation of all evaluation results from service requesters will constitute the overall evaluation of end user i regarding collaborative service device j, known as direct trust. Since trust is a dynamic value that changes over time, direct trust can be represented as $D_{i,j}^{t}$, as shown in Equation (2).

(2) $$D_{i,j}^{t}=\left|S_{i,j}^{t}\right|-\left|DS_{i,j}^{t}\right|$$

$\left|S_{i,j}^{t}\right|$ and $\left|DS_{i,j}^{t}\right|$ represent the number of satisfied and unsatisfied services provided by collaborative service device j to service-requesting end user i at time t, respectively.

In trust mechanisms, establishing trust typically requires multiple trusted interactions among participants; however, a single untrusted interaction can destroy the trust relationship. To describe these facts, this paper introduces a personalized context-aware evaluation mechanism. In this mechanism, a weight parameter $w\;\in \left[0,1\right]$ is defined to represent the impact of positive evaluations on direct trust, aiming to amplify the effect of unsatisfied services on direct trust. This mechanism effectively addresses the malicious behavior in which a collaborative service device initially provides effective services to gain high trust, only to deliver invalid services afterward. Under this mechanism, the direct trust $D_{i,j}^{t}$ of end user i towards cooperative service device j at time t can be expressed by Equation (3).

(3) $$D_{i,j}^{t}=w\;\times \left|S_{i,j}^{t}\right|-\left|DS_{i,j}^{t}\right|$$

2. The Decay of Trust Value

Trust is often closely related to time, as outlined in its characteristics [21]. During the period when the collaborative service device is providing services, it may have a relatively high trust value. However, this does not guarantee that it will maintain a high trust value in the present or future. Over time, the influence of user feedback will gradually decrease [22]. To account for this phenomenon, this paper introduces an attenuation factor. Consequently, the numbers of satisfied and unsatisfied services for end user i at time $t_{n}$ are represented by Equations (4) and (5), respectively.

(4) $$\left|S_{i,j}^{t}\right|=\left|S_{i,j}^{t_{n}}\right|+\sum_{k=0}^{n-1}e^{-\left(t_{f}-t_{k}\right)}\left|S_{i,j}^{t_{k}}\right|$$

(5) $$\left|DS_{i,j}^{t}\right|=\left|DS_{i,j}^{t_{n}}\right|+\sum_{k=0}^{n-1}e^{-\left(t_{f}-t_{k}\right)}\left|DS_{i,j}^{t_{k}}\right|$$

Here, $t_{n}$ represents the current time, $t_{f}$ denotes the evaluation feedback time, and $t_{k}$ signifies the duration from the evaluation feedback to the current time, with the conditions $t_{n}\geq t_{f}$ and $t_{0}=t_{f}$.The function $e^{-\left(t_{f}-t_{k}\right)}\in \left(0,1\right)$ denotes the decay factor. Therefore, the direct trust $D_{i,j}^{t_{n}}$ of end user i towards cooperative service device j at time j can be expressed as Equation (6).

(6) $$D_{i,j}^{t_{n}}=w\;\times \left(\left|S_{i,j}^{\left(t_{n}\right)}\right|+\sum_{k=0}^{n-1}e^{-\left(t_{f}-t_{k}\right)}\left|S_{i,j}^{t_{k}}\right|\right)-\left(\left|DS_{i,j}^{t_{n}}\right|+\sum_{k=0}^{n-1}e^{-\left(t_{f}-t_{k}\right)}\left|DS_{i,j}^{t_{k}}\right|\right)$$

3. Trust Value Update

The trust mechanism proposed in this paper updates the trust values of collaborative service devices after they complete their services. This aims to encourage effective collaborative services and discourage ineffective service behaviors. The updated trust value serves as the basis for the next selection. After the service is completed, the trust value of a cooperative service device that provides effective service may increase according to Equation (7). After the service is completed, the trust value of a cooperative service device that provides ineffective service may decrease according to Equation (8).

(7) $$D_{i,j}^{t_{n}}=D_{i,j}^{t_{n}}+\varsigma \times D_{i,j}^{t_{n}-1}$$

(8) $$D_{i,j}^{t_{n}}=\left\{\begin{array}{cc} D_{i,j}^{t_{n}}-\sigma \times D_{i,j}^{t_{n}-1}\;\;\;if\; & D_{i,j}^{\left(t_{n}\right)}>\sigma \times D_{i,j}^{t_{n}-1}\\ 0 & otherwise \end{array}\right.$$

Here, $\varsigma \in \left[0,1\right]$ stands for reward factor. $\sigma \in \left[0,1\right]$ stands for penalty factor and ς>σ. The reward factor and penalty factor are typically set by the edge service provider.

4. Normalization

To prevent the false inflation of direct trust caused by mutual collusion between malicious end users and collaborative service devices, this paper normalizes the direct trust values between end users and collaborative service devices, as shown in Equation (9).

(9) $$c_{i,j}^{t_{n}}=\left\{\begin{array}{cc} \frac{max\left(0,D_{i,j}^{t_{n}}\right)}{\sum_{j}max\left(0,D_{i,j}^{t_{n}}\right)} & if\sum_{j}max\left(0,D_{i,j}^{t_{n}}\right)>0\\ p_{j} & otherwise \end{array}\right.$$

Here, $c_{i,j}^{t_{n}}$ represents the normalized direct trust of terminal user i towards collaborative service device j at time $t_{n}$. If a set P of trustworthy collaborative service devices is known in advance—that is, every device in P is trustworthy—then for the scenario where terminal user i has not interacted with other collaborative service devices, $\sum_{j}max\left(0,D_{i,j}^{t_{n}}\right)=0$ is obtained. In this case, if collaborative service device j belongs to the trustworthy set P, then $p_{j}=1/\left|P\right|$ applies; otherwise, $p_{j}=0$ is used. If the trustworthy set P cannot be obtained in advance, then $p_{j}=1/K$ applies, where K is the total number of collaborative service devices.

Based on the above discussion, the direct trust value calculation method is presented in Algorithm 1.
**Algorithm 1** Direct Trust Value Calculation Algorithm**Input:** Quantity of interactions N, end user assessment of each service $tr\left[1,2,\cdots ,N\right]$,
coefficient of weight w, the current moment $t_{n}$ when the trust value is computed, assess the feedback message timing $t_{f}$, reward coefficient ς, penalty coefficient σ.

**Output:** The direct trustworthiness value $c_{i,j}^{t_{n}}$ of the end user, service satisfaction evaluation set S, evaluation set DS of service dissatisfaction, quantity of service satisfaction ratings tr_pos, Quantity tr_neg of service evaluations that are unsatisfactory.

**1:** tr_pos=tr_neg=0, S←Ø, DS←Ø;

**2:** if $t_{n}=t_{f}$
**then**

**3:**  for k←1
**to**
N
**do**

**4:**    if $tr\left[k\right]<0$
**then**

**5:**     $DS\leftarrow DS\cap tr\left[k\right]$;
**6:**     tr_neg++;

**7:**    **else**

**8:**     $S\leftarrow S\cap tr\left[k\right]$;
**9:**     tr_pos++;

**10:**   **end if**

**11:**   $D_{i,j}^{t_{n}}=\left(w\times tr\_pos-tr\_neg\right)/N$

**12:** **end for**

**13:**   **if** the provision of cooperative service devices constitutes a valid service. **then**

**14:**    Update the direct trust value $c_{i,j}^{t_{n}}$ in accordance with Equation (7).

**15:**   **else**

**16:**   Update the direct trust value $c_{i,j}^{t_{n}}$ in accordance with Equation (8).

**17:**   **end if**

**18:** **end if**

**19:**  Determine $c_{i,j}^{t_{n}}$ in accordance with Equation (9).
**20:** **if** $t_{n}>t_{f}$
**then**

**21:** Determine $D_{i,j}^{t_{n}}$ in accordance with Equation (6).

**22:**  **if** cooperative service devices offer efficient services. **then**

**23:**   Update the direct trust value $c_{i,j}^{t_{n}}$ in accordance with Equation (8).

**24:**  **else**

**25:**   Update the direct trust value $c_{i,j}^{t_{n}}$ in accordance with Equation (9).

**26:**  **end if**

**27:** **end if**

**28:**  Update the direct trust value $c_{i,j}^{t_{n}}$ in accordance with Equation (9).

**29: return**
tr_pos, tr_neg, S, DS, $c_{i,j}^{t_{n}}$

#### 3.2.2. Objective Indirect Trust

Upon completion of the task by the collaborative service device, the end user’s direct trust feedback is submitted to the edge service provider. Consequently, the edge service provider can construct an N×N matrix $C=\left[c_{i,j}^{t}\right]$ to represent the normalized direct trust values between any terminal user and any collaborative service device at time t. The Markov matrix is presented in Equation (10).

(10) $$C=\left[\begin{array}{ccccc} c_{1,1}^{t} & \ldots & c_{1,j}^{t} & \ldots & c_{1,N}^{t}\\ \ldots & \ldots & \ldots & \ldots & \ldots \\ c_{i,1}^{t} & \ldots & c_{i,j}^{t} & \ldots & c_{i,N}^{t}\\ \ldots & \ldots & \ldots & \ldots & \ldots \\ c_{N,1}^{t} & \ldots & c_{N,j}^{t} & \ldots & c_{N,N}^{t} \end{array}\right]$$

Direct trust represents the trust relationship established between end users and collaborative service devices based on their mutual interactions. In FIoT edge computing systems with end-to-edge collaboration mechanisms, interactions are influenced by interaction history and the mobility of end users. As a result, interactions often occur between parties who are not familiar with each other. When there is no interaction between end users and collaborative service devices, direct feedback information is unavailable. In such cases, it is necessary to utilize trust values provided by other devices that have interacted with the collaborative service device to assess its trustworthiness. This trust value is referred to as the objective indirect trust value in this paper.

In real-world interactions, people tend to trust information from those they know and trust. Similarly, when calculating indirect trust in this paper, we reference the trust values of neighboring devices with higher trust levels. Trust is often transitive in human society [23]. Similarly, in the proposed trust mechanism, if terminal user i trusts device j, and device j trusts device k, then even without direct interaction between user i and device k, trust can be transferred. This concept is formally described using Equation (11).

(11) $$I_{i,k}^{t}=\sum_{j}c_{i,j}^{t}\times c_{j,k}^{t}$$

Indirect trust in collaborative service devices can be established by aggregating the direct trust values from all their neighbors. Terminal users calculate indirect trust to collaborative service devices by considering only their neighbors, a process referred to as one-hop trust propagation in this paper. We introduce one vector $\vec{c_{i}^{t}}=\left[c_{i,j}^{t}\right]$ to represent the normalized direct trust of terminal users to all collaborative service devices and another vector $\vec{I_{i}^{t}}=\left[I_{i,j}^{t}\right]$ to represent the indirect trust. Therefore, the one-hop trust propagation can be represented in matrix form as shown in Equation (12).

(12) $$\vec{I_{i}^{t}}=C^{T}\times \vec{c_{i}^{t}}$$

In FIoT edge computing systems with sparse evaluation data, indirect trust obtained through one-hop trust transfer often cannot meet the interaction needs of end users. To gain a broader trust perspective, end user i can consider the evaluation information of its neighbors’ neighbors, known as two-hop trust transfer. Two-hop trust transfer can be formally expressed as Equation (13).

(13) $$\vec{I_{i}^{t}}={\left(C^{T}\right)}^{2}\times \vec{c_{i}^{t}}$$

Similarly, M-hop trust transfer can be expressed as the Equation (14).

(14) $$\vec{I_{i}^{t}}={\left(C^{T}\right)}^{m}\times \vec{c_{i}^{t}}$$

To address inaccurate neighbor evaluations, this paper introduces evaluation similarity. Evaluation similarity generally refers to the degree of similarity between two people’s views on the same thing. In our indirect trust calculations, evaluation credibility refers to how trustworthy the trust evaluations of cooperative service devices are. Given end user i and neighbor k, we use $r_{i,k}^{t}$ to represent the evaluation credibility of neighbor k from the perspective of end user i. The credibility is shown in Equation (15).

(15) $$r_{i,k}^{t}=\left\{\begin{array}{cc} \frac{\sum_{k\in \Xi_{i,k}^{t}}c_{i,o}^{t}\times c_{o,k}^{t}}{\sqrt{\sum_{k\in \Xi_{i,k}^{t}}{\left(c_{i,o}^{t}\right)}^{2}}\times \sqrt{\sum_{k\in \Xi_{i,k}^{t}}{\left(c_{o,k}^{t}\right)}^{2}}} & if\left|\Xi_{i,k}^{t}\right|>0\\ 0.5 & otherwise \end{array}\right.$$

Here, $\Xi_{i,k}^{t}$ represents the set of cooperative service devices that have interacted with both end user i and neighbor k.

From Equation (15), we infer that the more similar terminal user j’s evaluations are to those of terminal user i, the more reliable user j’s evaluation information is. By further considering the credibility of terminal user evaluation $r_{i,j}^{t}$, the direct trust $D_{i,j}^{t}$ between terminal users and collaborative service devices can be rewritten as shown in Equation (16).

(16) $$D_{i,j}^{t}=r_{i,j}^{t}\times D_{i,j}^{t}$$

Through the normalization operation of Equation (7) and the trust transfer operation of Equation (14), a more accurate end user indirect trust vector $\vec{I^{t}}$ can be obtained.

The indirect trust calculation algorithm is presented in Algorithm 2.
**Algorithm 2** Indirect Trust Value Computation Algorithm**Input:** Set A of cooperative service devices, the direct trust value calculated by Algorithm 1, time t.

**Output:**
$\vec{I^{t}}\left(A\right)$

**1:** Constructing matrix CC.

**2:**  for j∈A
**do**

**3:**   Calculate $\vec{I^{t}}$ in accordance with Equation (14).

**4:**  **end for**

**5:**   **return**
$\vec{I^{t}}\left(A\right)$


#### 3.2.3. Aggregate Trust

From the above discussion, we can obtain both direct and indirect trust values. In order to improve the reliability of trust calculation, this paper proposes an adaptive method to calculate the aggregated trust value. The aggregated trust value is shown in Equation (17).

(17) $$G_{i,j}^{t}=\sigma D_{i,j}^{t}+\left(1-\sigma \right)I_{i,j}^{t}$$

where σ and 1−σ are the weight coefficients for the direct and indirect trust values, respectively. σ can be calculated by Equation (18).

(18) $$\sigma =\left\{\begin{array}{cc} 1-e^{-N_{i,j}^{t}} & if\;\;\;\;\sum_{i=1}^{\left|A\right|}D_{i}^{t}/\left|A^{t}\right|\geq 0.5\\ 1 & \;\;if\;\;\;\;\sum_{i=1}^{\left|A\right|}D_{i}^{t}/\left|A^{t}\right|<0.5\\ 0 & if\;\;\;\;no\;direct\;exchange\;behavior \end{array}\right.$$

where $\sum_{i=1}^{\left|A\right|}D_{i}^{t}/\left|A^{t}\right|$ represents the average direct trust value of devices in the collaborative service device set at time t. $N_{i,j}^{t}$ represents the total number of successful interactions between end user i and cooperative service device j at time t.

From Equation (18), when there are no direct interactions at time t, σ is set to 0, and only the indirect trust value is used. When the average direct trust value in the collaborative service device set at time t is less than 0.5, it indicates that the direct trust is inaccurate, or the number of devices is small. To improve the credibility of the trust value, σ is set to 1; only the direct trust value is used, and the indirect trust value is ignored. When the average direct trust value of devices is greater than or equal to 0.5, the aggregated trust value is determined by considering both direct and indirect trust.

In summary, the aggregate trust computation algorithm is shown in Algorithm 3.
**Algorithm 3** Aggregate Trust Value Calculation Algorithm**Input:** Time t, Set A of cooperative service devices.

**Output:**
$G_{i,j}^{t}$

**1:** Determine $c_{i,j}^{t_{n}}$ in accordance with Algorithm 1.

**2:** Determine $\vec{I^{t}}\left(A\right)$ in accordance with Algorithm 2.

**3:** Calculate the mean value of direct trust in A.

**4:** $\sigma =\left\{\begin{array}{cc} 1-e^{-N_{i,j}^{t}} & if\;\;\;\;\sum_{i=1}^{\left|A\right|}D_{i}^{t}/\left|A^{t}\right|\geq 0.5\\ 1 & \;\;if\;\;\;\;\sum_{i=1}^{\left|A\right|}D_{i}^{t}/\left|A^{t}\right|<0.5\\ 0 & if\;\;\;\;no\;direct\;exchange\;behavior \end{array}\right.$

**5:** $G_{i,j}^{t}=\sigma D_{i,j}^{t}+\left(1-\sigma \right)I_{i,j}^{t}$

**6:**  **return**
$G_{i,j}^{t}$


### 3.3. Trusted Cooperative Service Device Selection Mechanism

In selecting trustworthy collaborative service devices, direct or indirect trust is typically used for screening. Several existing algorithms consider only the indirect trust values of collaborative service devices, neglecting their personalized direct trust. Therefore, after introducing the black- and whitelist mechanism, this paper proposes a trustworthy collaborative service device selection mechanism based on it. The personalized black- and whitelist mechanism filters out most malicious behaviors by prioritizing interaction with devices on the whitelist and avoiding those on the blacklist. This aims to enhance service quality in collaborative edge computing systems.

#### 3.3.1. Black- and Whitelist Mechanism

The presence of malicious acts significantly challenges the trustworthiness and accuracy of trust. If malicious devices exploit the model for attacks, it will undoubtedly diminish the trustworthiness and accuracy of trust. Therefore, we propose adopting a black- and whitelist mechanism to filter cooperative service devices. The black- and whitelist mechanism includes a blacklist, a whitelist, and a conversion mechanism between them.

1. Blacklist Mechanism

In the personalized black- and whitelist mechanism, end user i adds untrustworthy cooperative service devices to the blacklist and ceases interaction with them. Let B represent the blacklist of end user i. The blacklist mechanism is influenced not only by the number of satisfied and unsatisfied services but also by tolerance. Next, we introduce the definition of tolerance and the calculation process of the blacklist mechanism.

Tolerance $\delta_{i,j}^{t}\in \left[0,1\right]$ indicates the extent to which end users tolerate unsatisfactory services. The greater the value of $\delta_{i,j}^{t}$ is, the higher the tolerance level of end users is. In sociology, tolerance is jointly influenced by satisfaction and service fairness. Similarly, we introduce satisfaction and service fairness of cooperative service devices as two influencing factors. As discussed earlier, the satisfaction level $s_{i,j}^{t}$ of end users with services provided by cooperative service devices can be expressed by Equation (19).

(19) $$s_{i,j}^{t}=\frac{\left|S_{i,j}^{t}\right|}{\left|S_{i,j}^{t}\right|+\left|DS_{i,j}^{t}\right|}$$

Service fairness is also subjectively evaluated by the end user after the service is completed. Let $\Gamma =\left\{g\left|g\in \left[-2,2\right]\right.,g\in Ζ\right\}$ represent the set of evaluations. Each evaluation result $tl\left(i,j\right)$ belongs to the set Γ. The multi-dimensional evaluation mechanism can be expressed by Equation (20).

(20) $$tl\left(i,j\right)=\left\{\begin{array}{cc} 2 & very\;fair\\ 1 & fair\\ 0 & general\;case\\ -1 & unfair\\ -2 & very\;unfair \end{array}\right.$$

The service fairness evaluation from end user i to cooperative service device j is formed by aggregating all evaluation results from service requesters. Therefore, the service fairness $\vartheta_{i,j}^{t}$ of the collaborative service device can be expressed by Equation (21).

(21) $$\vartheta_{i,j}^{t}=\frac{\left|F_{i,j}^{t}\right|}{\left|F_{i,j}^{t}\right|+\left|DF_{i,j}^{t}\right|}$$

Here, $\left|F_{i,j}^{t}\right|$ and $\left|DF_{i,j}^{t}\right|$ represent the numbers of services evaluated as fair and unfair by end user i for cooperative service device j, respectively. Thus, the end user tolerance $\delta_{i,j}^{t}$ can be expressed by Equation (22).

(22) $$\delta_{i,j}^{t}=r\;\times \vartheta_{i,j}^{t}+\left(1-r\;\right)\times s_{i,j}^{t}$$

Here, $r\;\in \left[0,1\right]$ is a weight coefficient, generally set by the edge service provider. The tolerance is calculated as shown in Algorithm 4.
**Algorithm 4** The Algorithm for Tolerance Calculation
**Input:** The quantity of interactions N, the evaluation value $tr\left[1,2,\cdots ,N\right]$ of each service as perceived by the end user, the fairness rating $tl\left[1,2,\cdots ,N\right]$ of each service as perceived by the end user, the factor of service fairness r.

**Output:** Tolerance $\delta_{i,j}^{t}$

**1:** tr_pos=tr_neg=tl_pos=tl_neg=0, S←Ø, DS←Ø, F←Ø, FS←Ø;

**2:**  **for** k←1
**to** N
**do**

**3:**   **if** $tr\left[k\right]\geq 0$ **then**

**4**:   $S\leftarrow S\cap tr\left[k\right]$;

**5:**   tr_pos++;

**6:**   **else**

**7:**   $DS\leftarrow DS\cap tr\left[k\right]$;
**8:**   tr_neg++;

**9:**   **end if**

**10:**   **if** $tl\left[k\right]\geq 0$
**then**

**11:**   $F\leftarrow F\cap tl\left[k\right]$;

**12:**   tl_pos++;

**13:**   **else**

**14:**   $DF\leftarrow DF\cap tl\left[k\right]$;

**15:**   tr_neg++;

**16:**   **end if**

**17:**  **end for**

**18:**  $\delta_{i,j}^{t}=r\;\times \frac{tl\_pos}{tl\_pos+tl\_neg}+\left(1-r\;\right)\times \frac{tr\_pos}{tr\_pos+tr\_neg}$

**19: return** $\delta_{i,j}^{t}$

2. Construction of Blacklist

Based on the numbers of satisfactory and unsatisfactory services and the tolerance introduced earlier, terminal users will add malicious devices to the blacklist with a certain probability. The probability $pb_{i,j}^{t}$ that end user i adds cooperative service device j to the blacklist can be expressed by Equation (23).

(23) $$pb_{i,j}^{t}=\frac{1}{1+e^{\left(1-x_{i,j}^{t}\right)\delta_{i,j}^{t}}}$$

Here, $x_{i,j}^{t}=\left|DS_{i,j}^{t}\right|-\left|S_{i,j}^{t}\right|$, $\left|S_{i,j}^{t}\right|$, and $\left|DS_{i,j}^{t}\right|$ represent the numbers of satisfactory and unsatisfactory services provided by cooperative service device j to end user i, respectively. $\delta_{i,j}^{t}$ indicates the tolerance level of the end user for unsatisfactory services.

3. Whitelist Mechanism

In the personalized black- and whitelist mechanism, end user i adds trustworthy collaborative service devices to the whitelist and interacts with them. Let W represent the whitelist of end user i. The whitelist mechanism is influenced not only by the number of satisfactory and unsatisfactory services but also by the degree of acceptance. Next, we present the definition of acceptance and the calculation process of the whitelist mechanism.

In this paper, the acceptance rate $\eta_{i,j}^{t}\in \left[0,1\right]$ represents the degree to which end users acknowledge the satisfaction of the provided service. The larger the value of $\eta_{i,j}^{t}$ is, the higher the degree of end user acceptance is. In sociology, acceptance rate is influenced by both satisfaction and preference; thus, we introduce these two factors for cooperative service devices. The end users’ degree of satisfaction with the services provided by cooperative service devices is expressed by Equation (19). The preference degree of end user i for device j, denoted as $\nu_{i,j}$, is defined as the percentage of successful interactions between them, as shown in Equation (24). The edge service provider calculates and aggregates the number of interactions.

(24) $$\nu_{i,j}^{t}=\frac{N_{i,j}^{s\left(t\right)}}{\sum_{k=1}^{M}N_{k}^{t}}$$

Here, $N_{i,j}^{s\left(t\right)}$ represents the number of successful interactions between end user i and device j; $\sum_{k=1}^{M}N_{k}^{t}$ represents the total number of interactions of end user i; M represents the total number of devices providing services to end user i. Given a fixed total number of interactions, an increase in interactions with a specific device indicates a preference for that device. Therefore, the degree of acceptance of satisfactory services by end user i can be expressed by Equation (25).

(25) $$\eta_{i,j}^{t}=\kappa \times v_{i,j}^{t}+\left(1-\kappa \right)\times s_{i,j}^{t}$$

Here, $\kappa \in \left[0,1\right]$ is a weight coefficient, generally set by the edge service provider.

4. Whitelist Mechanism

Based on the numbers of satisfactory and unsatisfactory services and the acceptance rate discussed earlier, end users will add devices with honest behavior to the whitelist with a certain probability. The probability $pw_{i,j}^{t}$ that end user i adds device j to the whitelist is given by Equation (26).

(26) $$pw_{i,j}^{t}=\frac{1}{1+e^{\left(1+x_{i,j}^{t}\right)\eta_{i,j}^{t}}}$$

Here, $x_{i,j}^{t}=\left|DS_{i,j}^{t}\right|-\left|S_{i,j}^{t}\right|$, $\left|S_{i,j}^{t}\right|$ and $\left|DS_{i,j}^{t}\right|$ represent the numbers of satisfactory and unsatisfactory services provided by device j to end user i, respectively. $\eta_{i,j}^{t}$ represents the degree of acceptance by end user i.

#### 3.3.2. Dynamic Whitelist Conversion Mechanism

In a personalized whitelist mechanism, the behavior of collaborative service devices constantly changes. Devices on the whitelist may exploit their high trust value to deliver invalid or malicious services. Devices on the blacklist may provide good services to improve their trust value. To capture the dynamic behavior of devices, we propose a dynamic transformation mechanism for the personalized whitelist, consisting of a whitelist removal mechanism and a blacklist removal mechanism.

1. Blacklist and Whitelist Removal Mechanism

If device j is on the whitelist of end user i and end user i receives an invalid or malicious service from it, device j will be moved from the whitelist to the normal user list with probability $pw_{i,j}^{m\left(t\right)}$. The probability $pw_{i,j}^{m\left(t\right)}$ is related to the following four attributes:

(1) The ratio $\frac{\left|S_{i,j}^{t}\right|}{\left|S_{i,j}^{t}\right|+\left|DS_{i,j}^{t}\right|}$ of unsatisfactory services to total services. A larger $\frac{\left|S_{i,j}^{t}\right|}{\left|S_{i,j}^{t}\right|+\left|DS_{i,j}^{t}\right|}$ indicates that device j provides invalid services more frequently, increasing the probability of its removal from the whitelist.
(2) To prevent device j from strategically providing unsatisfactory services while maintaining a high effective service ratio, we consider the number of ineffective services $\left|DS_{i,j}^{t}\right|$ provided by device j. The larger $\left|DS_{i,j}^{t}\right|$ is, the higher the probability is of removing device j from the whitelist.
(3) The importance of the service: If collaborative service device j provides false feedback on highly important services, the probability of removing device j from the whitelist increases; conversely, for less important services, this probability decreases.
(4) Terminal user i’s sensitivity to ineffective or malicious services $\phi_{i}\in R$: A larger $\phi_{i}$ value indicates that user i has less tolerance for ineffective or malicious services, increasing the probability of removing device j. Conversely, a smaller $\phi_{i}$ decreases this probability. Therefore, $pw_{i,j}^{m\left(t\right)}$ is defined as shown in Equation (27).

(27) $$pw_{i,j}^{m\left(t\right)}=\frac{2}{1+e^{-\phi_{i}\times \left|DS_{i,j}^{t}\right|\times \frac{\sum_{\chi \in DS_{i,j}^{t}}DI\left(\chi \right)}{\sum_{\chi \in DS_{i,j}^{t}}DI\left(\chi \right)+\sum_{\Psi \in S_{i,j}^{t}}DI\left(\Psi \right)}}}-1$$

Specifically, $DS_{i,j}^{t}$ represents the set of transactions where terminal user i is dissatisfied with services from equipment j at time t, while $S_{i,j}^{t}$ represents the set where user i is satisfied. Here, χ is a single unsatisfactory service between devices, Ψ is a single satisfactory service, and the function $DI\left(•\right)$ represents the importance of the service.

2. Blacklist Deletion Mechanism

Suppose that cooperative service device j is on the blacklist of end user i. Considering that device j may improve its untrustworthy behavior to enhance its trustworthiness, end user i will periodically calculate the probability $pb_{i,j}^{m\left(t\right)}$ of removing device j from the blacklist. The probability $pb_{i,j}^{m\left(t\right)}$ depends on the following three attributes:

(1) Neighbor k’s attitude ${ϒ}_{k,j}^{t}$ towards device j: ${ϒ}_{k,j}^{t}=a_{1}$ denotes that neighbor k has blacklisted device j; ${ϒ}_{k,j}^{t}=a_{2}$ indicates placement on the regular list; ${ϒ}_{k,j}^{t}=a_{3}$ indicates the whitelist, with $a_{1},a_{2},a_{3}\in N_{+}$ and $a_{3}>a_{2}>a_{1}$ defined accordingly. The more favorable neighbor k’s attitude is towards device j, the higher the probability is that end user i will remove j from the blacklist.
(2) Evaluation reliability $r_{i,k}^{t}$ between end user i and neighbor k: A higher $r_{i,k}^{t}$ means neighbor k has a greater influence on $pb_{i,j}^{m\left(t\right)}$.
(3) End user i’s aversion to invalid or malicious services $\xi_{i,j}^{t}$: A higher value $\xi_{i,j}^{t}$ makes it less likely for device j to be removed from the blacklist. In summary, $pb_{i,j}^{m\left(t\right)}$ is defined as shown in Equation (28).

(28) $$pb_{i,j}^{m\left(t\right)}=\left\{\begin{array}{c} 1\;if\;\sum_{k\in N_{i}}\frac{{ϒ}_{k,j}^{t}\times r_{i,k}^{t}}{\left|N_{i}\right|}\geq \xi_{i,j}^{t}\\ 0\;\;\;otherwise \end{array}\right.$$

Here, $N_{i}$ represents the set of neighbor devices of terminal user i. The aversion degree $\xi_{i,j}^{t}$ can be obtained through statistical analysis of user i’s history, calculated by Equation (29).

(29) $$\xi_{i,j}^{t}=1-e^{-\sum_{h=1}^{\left|DS\right|}tr[h]}$$

In summary, the dynamic mechanism for converting between blacklists and whitelists is presented in Algorithm 5.

Based on the personalized whitelist and dynamic transfer mechanisms, the proposed collaborative service device selection algorithm is presented in Algorithm 6.
**Algorithm 5** Black- and whitelist dynamic conversion mechanism**Input:** end user i’s blacklist $B_{i}$, end user i’s whitelist $W_{i}$ general list R

**Output:** end user i ’s blacklist after conversion ${B^{\prime }}_{i}$, end user i’s whitelist after conversion ${W_{i}}^{\prime }$, general after conversion $R^{\prime }$

**1:**
${B^{\prime }}_{i}\leftarrow B_{i}$, ${W^{\prime }}_{i}\leftarrow W_{i}$, $R^{\prime }\leftarrow R$

**2:** **if**
$W_{i}\neq Ø$
**then**

**3:**    $pw_{i,j}^{m}$ is calculated according to Equation (27).

**4:**  rmd=rand()

**5:**    **if**
$rmd\leq pw_{i,j}^{m}$

**6:**   ${W^{\prime }}_{i}\leftarrow W_{i}-\left\{i\right\}$

**7:**   ${R^{\prime }}_{i}\leftarrow R_{i}-\left\{i\right\}$

**8:**   **else**

**9:**    The co-serving device j continues to remain in the set $W_{i}$

**10:**  **end if**

**11: end if**

**12: if**
$B_{i}\neq Ø$
**then**

**13:**  $pb_{i,j}^{m}$ is calculated according to Equation (28).
**14:**  **if**
$pb_{i,j}^{m}=1$

**15:**    ${B^{\prime }}_{i}\leftarrow B_{i}-\left\{i\right\}$

**16:**    ${R^{\prime }}_{i}\leftarrow R_{i}-\left\{i\right\}$

**17:**  **else**

**18:**  The co-serving device j continues to remain in the set $B_{i}$

**19:**  **end if**

**20: end if**

**21: return**
${B^{\prime }}_{i}$, ${W_{i}}^{\prime }$, $R^{\prime }$

**Algorithm 6** Collaborative service device selection algorithm based on personalized black- and whitelist**Input:** end user i’s blacklist $B_{i}$, end user i’s whitelist $W_{i}$, the set R of cooperative service devices for the service request is named, the cumulative trust value $t_{k}$ of the cooperative service device k, k∈R

**Output:** The chosen cooperative service device j

**1:** L=Ø

**2:** $R^{\prime }\leftarrow R-B_{i}$

**3:** **if**
$R^{\prime }==Ø$

**4:**   **return** −1

**5:** **else**

**6:**    $L\leftarrow R^{\prime }\cap W_{i}$

**7:**     **if**
L≠Ø

**8:**     The cooperative service device j is selected for execution with a probability of $p_{j}=t_{j}/\sum_{k=0}^{k=\left|L\right|}t_{k}$.

**9:**     **else**

**10:**    The cooperative service device j is selected for execution with a probability of $p_{j}=t_{j}/\sum_{k=0}^{k=\left|R\right|}t_{k}$.

**11:**   **end if**

**12: end if**

### 3.4. Resource Allocation Mechanism of Trusted Collaborative Service Devices

After selecting collaborative service devices using the personalized whitelist- and blacklist-based selection algorithm, trustworthy service providers were identified. To address resource allocation among the selected service devices, this paper proposes a collaborative service device resource allocation mechanism. The proposed resource allocation mechanism includes two parts: grouping rules and a coalition game.

1. Grouping Rules

First, the collaborative service devices in the whitelist are grouped. The grouping condition is determined by a grouping function calculated from the degree of activity and the aggregated trust value.

2. Degree of Activity

For a given whitelist set N, let ζ denote the effective number of interactions of all cooperating service devices. Let $\zeta_{i}$ represent the effective number of interactions of cooperative service device i. Clearly, $\zeta_{i}\subset \zeta$. Therefore, the ratio of device i’s effective interactions to the total effective interactions in the whitelist is defined as the activity degree of device i. The degree of activity is shown in Equation (30).

(30) $$\iota_{i}=\frac{\zeta_{i}}{\zeta }$$

3. Aggregate Trust Values

The aggregated trust value $G_{i,j}^{t}$ is the cumulative value of the direct and indirect trust of the cooperative service device at time t. Details can be found in Algorithm 3.

In conclusion, the grouping function of cooperative service devices within the whitelist at time t is presented in Equation (31).

(31) $$g_{i}^{t}=\iota_{i}^{t}\times \tau +\left(1-\tau \right)\times G_{i,j}^{t}$$

Here, τ is the scaling factor, $\tau \in \left[0,1\right]$.

When the condition $0<g_{i}^{t}\leq \bar{\omega }$ is satisfied, device i is included in set ℑ; otherwise, it is added to set ℘, where $\bar{\omega }$ is a hyperparameter. Regarding devices in set ℘, since their effective interaction times and aggregated trust values are relatively high, the devices are sorted in descending order based on the grouping function values. Devices with higher values are allocated resources preferentially. For devices in set ℑ, resource allocation is carried out using a coalition game method.

4. Coalition game

The coalition game model in this paper is defined as a triple $G=\langle N^{t},U^{t},F^{t}\rangle$. The game players are the devices in set ℑ. Assuming there are M players, the set of players at time t is $N^{t}=\left\{n_{1}^{t},n_{2}^{t},\cdots ,n_{M}^{t}\right\}$. In the coalition game, players calculate their utility values based on Equation (15). The utility value of each coalition is the sum of the utility values of its members. The payoffs of all coalitions constitute the utility value set $U^{t}=\left\{u_{1}^{t},u_{2}^{t},\cdots ,u_{M}^{t}\right\}$ of the game. The utility function is given in Equation (32).

(32) $$U_{i}=-0.5*E_{i}-0.5*T_{i}$$

Among these parameters, $T_{i}$ denotes the delay, while $E_{i}$ represents the energy consumption. For detailed formulations regarding the calculation of delay and energy consumption, please refer to reference [24].

The coalition structure represents the set of coalitions formed by all sub-coalitions at time t. Players can form between 1 and M coalitions, depending on circumstances. The coalition structure is represented by set $F^{t}=\left\{f_{1}^{t},f_{2}^{t},\cdots ,f_{l}^{t}\right\}$, where $f_{i}^{t}$ indicates the sub-coalitions at time t. Subsequently, the concept of alliance gaming is adopted to form alliances among collaborative service devices. As the number of devices in an alliance grows, a device may have fewer available resources or weaker processing capacity compared to others. As a result, this device obtains less revenue and may leave the current alliance to form a new sub-alliance. Similar to reference [25], we define the coalition adjustment rules for collaborative service devices by establishing preference relations.

**Definition 1.**  *For a cooperative service device i within set ℑ, the preference relation ${≻}_{i}$ on the set of all coalitions it may form possesses completeness, reflexivity, and transitivity.*

**Proof of Definition 1.**  Since the objective of coalition selection for cooperative service devices in set ℑ is to maximize utility and ensure each device has a non-negative utility value, the preference relation in Equation (33) holds for any cooperative service device i where i∈ℑ.

(33) $${\mathbb{C}}_{j}^{t}{≻}_{i}{\mathbb{C}}_{k}^{t}\Leftrightarrow U^{t}\left({\mathbb{C}}_{j}^{t}\right)+U^{t}\left({\mathbb{C}}_{k}^{t}\backslash i\right)>U^{t}\left({\mathbb{C}}_{j}^{t}\backslash i\right)+U^{t}\left({\mathbb{C}}_{k}^{t}\right)$$

(34) $$s.t.\left\{\begin{array}{c} U_{l}^{t}\left({\mathbb{C}}_{j}^{t}\right)\geq 0,U_{l}^{t}\left({\mathbb{C}}_{j}^{t}\backslash i\right)\geq 0,\forall l\in \left\{U^{t}\left({\mathbb{C}}_{j}^{t}\backslash i\right)\right\}\\ U_{l}^{t}\left({\mathbb{C}}_{k}^{t}\right)\geq 0,U_{l}^{t}\left({\mathbb{C}}_{k}^{t}\backslash i\right)\geq 0,\forall l\in \left\{U^{t}\left({\mathbb{C}}_{k}^{t}\backslash i\right)\right\} \end{array}\right.$$

According to Equation (33), if the total utility becomes greater in sub-alliance ${\mathbb{C}}_{j}^{t}$ than in ${\mathbb{C}}_{k}^{t}$ when cooperative service device i joins ${\mathbb{C}}_{j}^{t}$ at time t, and no other device l in ${\mathbb{C}}_{j}^{t}$ or ${\mathbb{C}}_{k}^{t}$ receives a negative utility due to this change, then device i is more inclined to join ${\mathbb{C}}_{j}^{t}$. □

The main aim of forming a coalition of collaborative service devices within set ℑ is to achieve a high utility value. Each collaborative service device in the coalition game can decide whether to join or leave a specific coalition based on its preference relation and utility value.

**Definition 2.**  *Given the alliance set ${\mathbb{C}}^{t}=\left\{{\mathbb{C}}_{1}^{t},{\mathbb{C}}_{2}^{t},\ldots ,{\mathbb{C}}_{M}^{t}\right\}$ at time t, if cooperative service device i decides to withdraw from its current sub-alliance ${\mathbb{C}}_{k}^{t}\in {\mathbb{C}}^{t}$ and join another sub-alliance ${\mathbb{C}}_{j}^{t}\in {\mathbb{C}}^{t}$, resulting in a new alliance partition $\hat{{\mathbb{C}}^{t}}=\left\{{\mathbb{C}}_{k}^{t}\backslash \left\{i\right\},{\mathbb{C}}_{j}^{t}\cup \left\{i\right\}\right\}\cup \left\{{\mathbb{C}}^{t}\backslash \left\{{\mathbb{C}}_{j}^{t},{\mathbb{C}}_{k}^{t}\right\}\right\}$.*

**Proof of Definition 2.**  If, at time t, a coalition adjustment by a cooperative service device in set ℑ can significantly enhance the system’s overall utility and does not cause other devices’ utility to become negative, then this adjustment should be implemented. The goal of the coalition game is to determine a sub-coalition partition scheme that maximizes the total utility of cooperative service devices, rather than the utility of individual devices. Additionally, the coalition game ensures that each participating cooperative service device receives appropriate remuneration. □

**Definition 3.**  *At time t, if no sub-alliance adjustment rules are satisfied, the cooperative service devices and sub-alliances in set ℑ form a stable structure.*

**Proof of Definition 3.**  According to Definition 2, the alliance structure will continuously adjust and change. Through continuous iterative search, the alliance structure will keep adjusting until the stability rules are satisfied, resulting in the optimal solution for the overall system. It is evident that the optimal solution is the final stable alliance structure, corresponding to the final allocation scheme of cooperative service devices in set ℑ at time t. □

5. Resource Allocation Algorithm for Trusted Cooperative Service Devices

With the introduction of grouping rules and the coalition game, the resource allocation algorithm for trusted cooperative service devices is derived, as shown in Algorithm 7. When allocating resources, the proportional fairness criterion is used. Allocation plan ${\mathrm{OC}}^{*}$ satisfies proportional fairness; any other allocation plan oc does not satisfy that all members $\frac{{\mathrm{oc}}_{i}-{{\mathrm{OC}}^{*}}_{i}}{{{\mathrm{OC}}^{*}}_{i}}$ are positive at the same time. Ensure that the benefits obtained by each member of the alliance are proportional to their contribution or demand and avoid excessive exploitation or preferential treatment of some members.
**Algorithm 7** The Resource Allocation Algorithm for Trusted Cooperative Service Devices**Input:** Whitelist set, valid interaction records of collaborative service devices included in the whitelist, probability of being included in the whitelist, the maximal number of iterations Λ, $\bar{\omega }$

**Output:** Resource allocation outcomes for devices within the whitelist collection.

**1:** Calculate the value of the grouping function for each device within the whitelist set in accordance with Equation (31).

**2:** **for**
i∈W
**do**

**3:**  **if**
$g_{i}>\bar{\omega }$
**then**

**4:**   $℘\cup \left\{i\right\}$

**5:**  **else**

**6:**   $ℑ\cup \left\{i\right\}$

**7:**  **end if**

**8:** **end for**

**9:** Randomly initialize the structure of the coalition within the set ℑ and n=0.

**10:** **for** j∈ℑ
**do**

**11:**  **for** ${\mathbb{C}}_{a}\in \mathbb{C}$
**do**

**12:**   **if** adhere to the rules stipulated in Definition 2 **then**

**13:**  The coalition adjustment is conducted, a new coalition is formed, and the device joining sequence is recorded.

**14:**   **else**

**15:**    The coalition structure remains unaltered.

**16:**   **end if**

**17:**   n=n+1

**18:**   **if** Definition 3 remains valid or n>Λ
**then**

**19:**    **break**

**20:**   **end if**

**21:**  **end for**

**22:** **end for**

**23:** For the devices in the set ℘, the grouping function value is sorted in descending order by means of the quick sort algorithm to form the allocation result.

**24:** For the devices within the set ℑ, the device resources are allocated in accordance with the game outcomes of the coalition game. In the process of resource allocation, the proportional fairness criterion is used.
**25: return** whitelist of device resource allocation outcomes.

6. Convergence of the algorithm

**Theorem 1.**  *The cooperative service device resource allocation algorithm can guarantee to reach the final stable solution ${\mathbb{C}}^{t}$ in set ℑ, and this solution is composed of multiple disjoint coalitions.*

**Proof of Theorem 1.**  Each cooperative service device in set ℑ at time t can perform coalition adjustment operations and generate new sub-coalitions according to preference relations and coalition adjustment rules in Definition 3. According to the coalition adjustment rule, Equation (35) holds since it can be transformed from ${\mathbb{C}}_{a}^{t}$ to ${\mathbb{C}}_{a+1}^{t}$ only when the system utility is improved.

(35) $${\mathbb{C}}_{a}^{t}\to {\mathbb{C}}_{a+1}^{t}\Leftrightarrow U^{t}\left({\mathbb{C}}_{a}^{t}\right)<U^{t}\left({\mathbb{C}}_{a+1}^{t}\right)$$

Since the number of cooperative service devices in set ℑ is limited, the number of new schemes generated by each adjustment operation is also limited, so the cooperative service device resource allocation algorithm can ensure that the coalition reaches the final stable scheme ${\mathbb{C}}^{t}$. □

7. Algorithm stability

In order to analyze the stability of the final solution ${\mathbb{C}}^{t}$ obtained after convergence of the collaborative service device resource allocation algorithm, the definition of the Nash equilibrium was first given.

**Definition 4.**  *For an instant t coalition structure ${\mathbb{C}}^{t}=\left\{{\mathbb{C}}_{1}^{t},{\mathbb{C}}_{2}^{t},\cdots ,{\mathbb{C}}_{M}^{t}\right\}$, ${\mathbb{C}}^{t}$ is Nash equilibrium if and only if both pairs $\forall i\in {ℑ}^{t},i\in {\mathbb{C}}_{j}^{t},{\mathbb{C}}_{j}^{t}{≻}_{i}{\mathbb{C}}_{k}^{t}\cup \left\{i\right\}$ hold, where j≠k.*

**Theorem 2.**  *The final solution ${\mathbb{C}}^{t}$ obtained from the cooperative service device resource allocation algorithm in set ℑ is Nash equilibrium.*

**Proof of Theorem 2.**  The proof is carried out by contradiction, assuming that the final scheme obtained by the cooperative service device resource allocation algorithm in set ℑ is not Nash equilibrium. Then, there must exist at time t the cooperative service device $i\in {\mathbb{C}}_{j}^{t}$ in set ℑ, and the sub-coalition ${\mathbb{C}}_{k}^{t}\subset {\mathbb{C}}^{t}$ and j≠k such that ${\mathbb{C}}_{k}^{t}\cup \left\{i\right\}{≻}_{i}{\mathbb{C}}_{j}^{t}$. This will inevitably lead to a change in the coalition structure, which is contrary to the conclusion that ${\mathbb{C}}^{t}$ is the final scheme. Therefore, the final solution ${\mathbb{C}}^{t}$ obtained by the cooperative service device resource allocation algorithm in set ℑ must be the Nash equilibrium. □

8. Time Complexity of the algorithm

**Theorem 3.**  *The time complexity of the resource allocation algorithm for cooperative service devices is polynomial time.*

**Proof of Theorem 3.**  In each iteration, the resource allocation algorithm of the collaborative service device selects only one collaborative service device in set ℑ, and then calculates the total utility value of the selected collaborative service device in the current coalition and the potential adjustment to the coalition, respectively, and decides whether to perform coalition adjustment operation according to the preference relationship. If the coalition adjustment operation is determined, the selected cooperative service device is adjusted from its current coalition to the new sub-coalition. Since the number of coalition adjustment operations in each iteration is, at most, l, and the maximum number of iterations is n, the collaborative service device resource allocation algorithm has $O\left(l\times n\right)$ computational complexity in the worst case in set ℑ. The quicksort method is used to sort in the set ℘; assuming that there are h devices, then the worst-case time complexity of quicksort is $h^{2}$. In summary, the time complexity of the resource allocation algorithm for cooperative service devices is the polynomial time. □

## 4. Results and Discussion

### 4.1. Experimental Setting

The simulation models the random distribution of 1000 users within a coverage area of radius 1 km. Five predefined trusted collaborative service devices were used. Without loss of generality, we assumed all users had the same tolerance and acceptance. According to references [26,27], typically, $\delta_{i}=\eta_{i}=10$ is employed. The system contains 2000 different services. Initially, each end user has an average of 15 services. Without loss of generality, we set all service transactions to have the same level of importance. We introduced 1000 rounds of initial interactions to establish trust between end users and collaborative service devices. The busy ratios of collaborative devices (PCDs) were set at 10%, 20%, 40%, and 100%, representing idle, busy, highly busy, and extremely busy states of the IoT edge computing system, respectively.

We denote the number of service requests initiated by ordinary end users by num_trans end user, and the number of effective services they obtained by num_valid. In the experiments, the trust model’s performance was measured by the effective service acquisition rate, denoted as r=num_valid/num_trans. For convenience, we abbreviated bad-mouthing and good-mouthing attacks as Model A, ballot-stuffing attacks as Model B, and selective behavior attacks as Model C. Since bad-mouthing and good-mouthing attacks are similar, we classified them as one type. We considered the completion of simple tasks in a collaborative FIoT edge computing scenario. To ensure experimental authenticity, the network topology in our simulations was randomly generated. All experiments were run on a Windows 10 machine with an Intel Core i5-4590 3.3 GHz processor and 8 GB of memory.

The experimental setup is summarized in Table 1.

### 4.2. Model Validation

**Figure 2:**
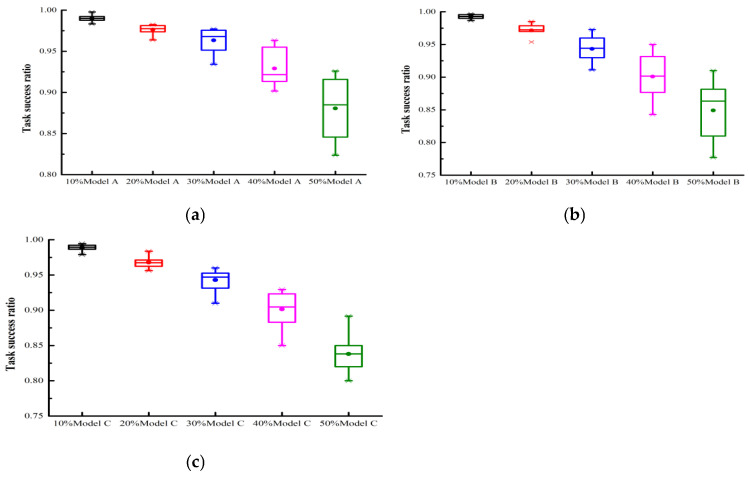
Task success ratio under different single attack models with PCD = 100%: (**a**) attack model A; (**b**) attack model B; (**c**) attack model C.

**Figure 3:**
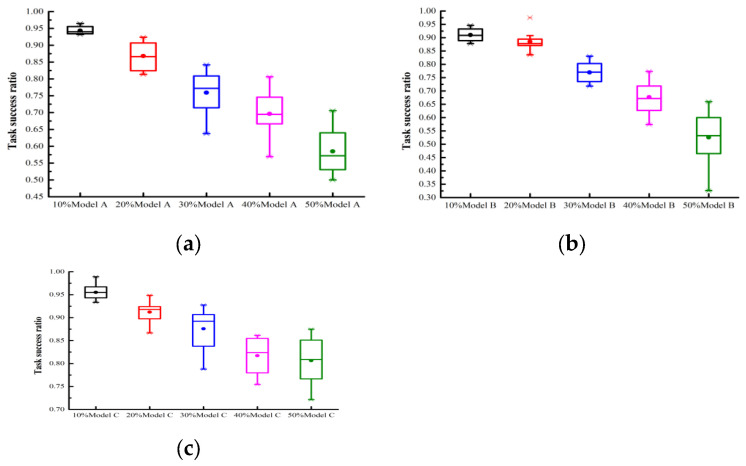
Task success ratio under different single attack models with PCD = 10%: (**a**) attack model A; (**b**) attack model B; (**c**) attack model C.

We evaluated the model’s performance under single attack behaviors. To evaluate the trust mechanism’s performance in ensuring quality of service, this paper focuses on: (1) diverse attack models from A to C; (2) malicious user proportions ranging from 10% to 50%; (3) measuring the effective service acquisition rate under three experimental settings with PCD values from 10% to 100%. In Figure 2 and Figure 3, the user collaboration degrees were set to 100% and 10%, respectively. The former represents an extremely busy FIoT edge computing system, while the latter indicates an idle system. Figure 2a examines the impact of attack model A under varying proportions of malicious users. The experimental results reveal that: (1) The accumulation of trust evaluation information allowed the proposed trust mechanism to precisely evaluate each user’s trust degree. Consequently, the system’s effective service acquisition rate gradually increased over time. Particularly, in scenarios with a high proportion of malicious users, the performance improvement was more pronounced. (2) In scenarios with 10%, 20%, 30%, 40%, and 50% malicious users, the proposed trust mechanism achieved effective service acquisition rates of 99.3%, 97.9%, 97.7%, 95.5%, and 92.6%, respectively, at the 10,000th interaction. These results demonstrate the proposed model’s effectiveness in guaranteeing the quality of service in collaborative FIoT edge computing systems. Figure 2b,c examine the impact of attack models B and C, respectively, under varying proportions of malicious users. Similar to Figure 2a, there was a gradual improvement in model performance over time. With 50% malicious users under attack models B and C, the proposed model achieved an effective service acquisition rate of approximately 90% after 10,000 interactions, demonstrating its robustness against different malicious attacks. In collaborative edge computing FIoT systems with a collaboration degree of 10%, the model could not accurately assess each user’s trustworthiness due to limited trust feedback. Consequently, as shown in Figure 3a–c, the system performance exhibited repeated oscillations over time, and gradual improvement could not be achieved. In systems with collaboration degrees of 20% and 40%, the model’s performance fell between PCD = 10% and PCD = 100%; therefore, it is not presented here to save space.

In FIoT scenarios, multiple types of malicious users may coexist. Therefore, to further investigate the effectiveness and robustness of the proposed trust model, we considered the influence of complex attacks involving multiple attack models on the trust mechanism’s performance. The experimental setup for the complex attack scenarios was as follows: (1) malicious user proportions were 10%, 20%, 30%, 40%, and 50%; (2) PCD values were 10%, 20%, 40%, and 100%; (3) to study the impact of different proportions of complex attack models on the task success rate, four complex attack scenarios were considered:

Scenario 1: Each malicious user randomly selected models A, B, or C with equal probability (1/3 each).

Scenario 2: Each malicious user selected models A, B, or C with probabilities of 15%, 60%, and 25%, respectively, making model B predominant.

Scenario 3: Each malicious user selected models A, B, or C with probabilities of 60%, 25%, and 15%, respectively, making model A predominant.

Scenario 4: Each malicious user selected models A, B, or C with probabilities of 25%, 15%, and 60%, respectively, making model C predominant.

**Figure 4:**
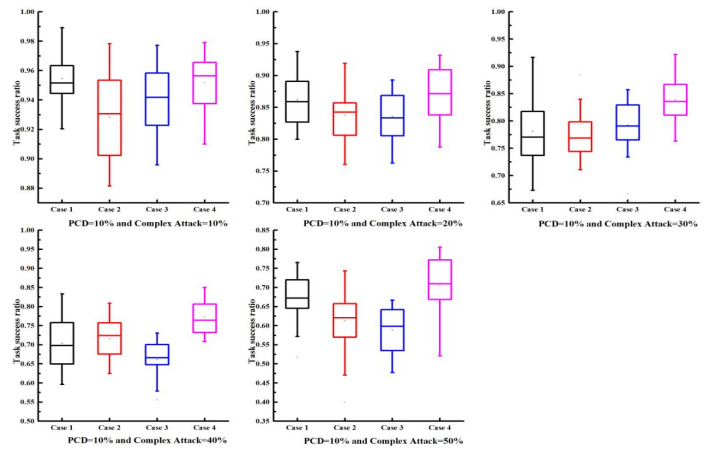
Task success ratio under different complex attack models with PCD = 10%.

**Figure 5:**
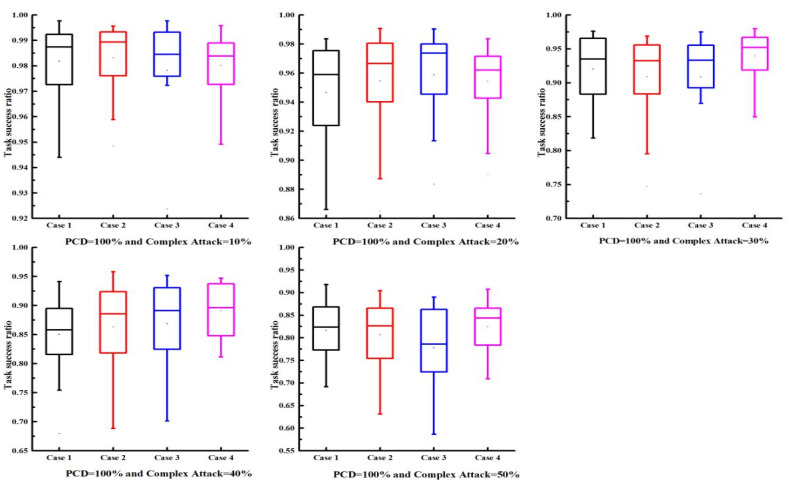
Task success ratio under different complex attack models with PCD = 100%.

The results are presented in Figure 4 and Figure 5. As the proportion of malicious users increased, the task success rate was expected to decline; however, the decline was relatively slow, suggesting that the trust model demonstrated robust performance. As PCD increased (i.e., more transaction data), the task success rate also increased. Particularly, when PCD = 100%, the average task success rate could reach 80% even with 50% malicious users, indicating the efficacy of the proposed model. The proposed trust model also achieved similar results under varying proportions of complex attack models, verifying its robustness against complex attacks. In conclusion, the proposed trust model maintained good performance even under complex attack scenarios.

**Figure 6:**
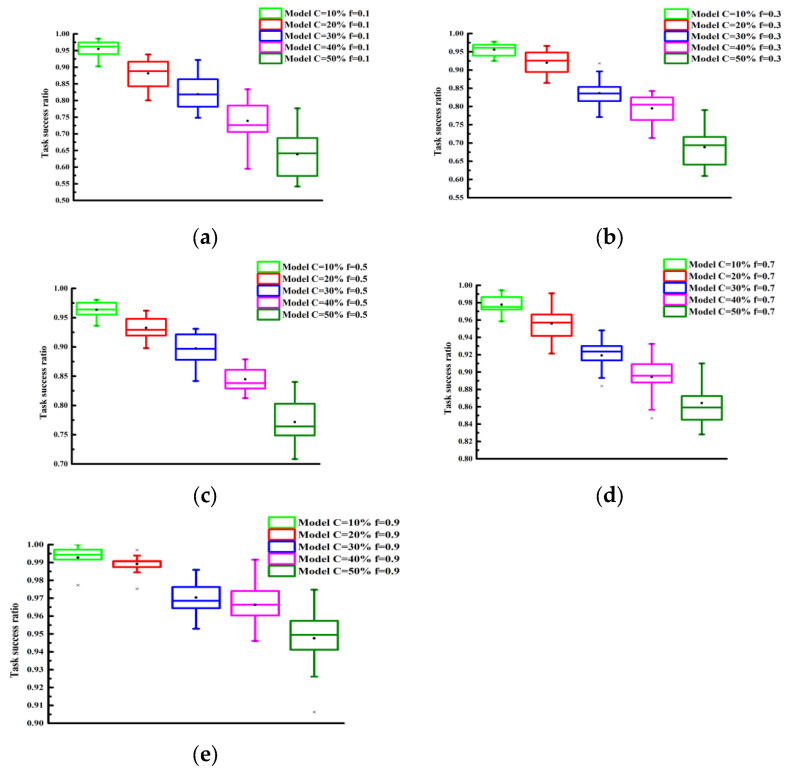
Task success ratio under different f values with PCD = 40%: (**a**) f=0.1 (**b**) f=0.3 (**c**) f=0.5 (**d**) f=0.7 (**e**) f=0.9.

**Figure 7:**
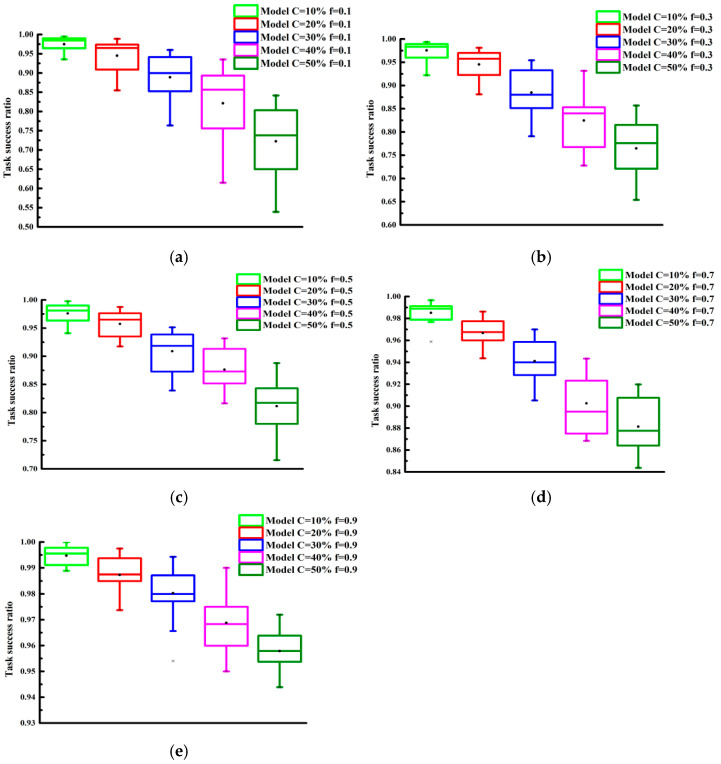
Task success ratio under different f values with PCD = 100%: (**a**) f=0.1 (**b**) f=0.3 (**c**) f=0.5 (**d**) f=0.7 (**e**) f=0.9.

The performance of the model under selective attack behaviors was examined. We further investigated the effect of selective aggression on the performance of the proposed trust model through experiments. The parameter f was varied from 10% to 90% in increments of 10%. The experimental results are presented in Figure 6 and Figure 7. As observed from the graphs, the task success rate increased as f increased. Additionally, Figure 6 and Figure 7 show that, for the same f value, with the more malicious nodes that were present, the lower the task success rate was.

### 4.3. Effectiveness of the Black- and Whitelist Mechanism

**Figure 8:**
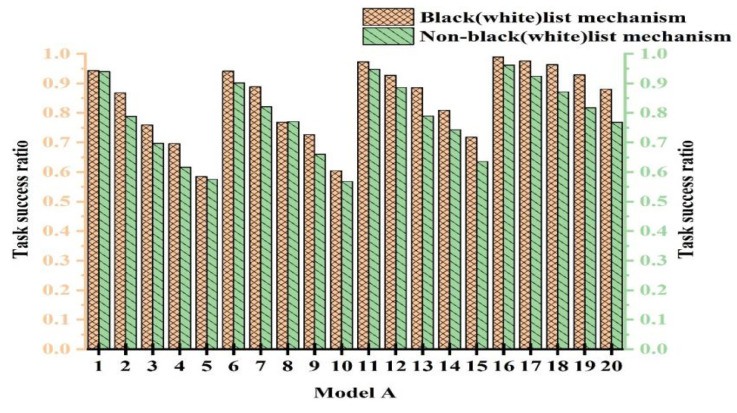
Task success ratio with the black- and whitelist mechanism under Attack Model A.

**Figure 9:**
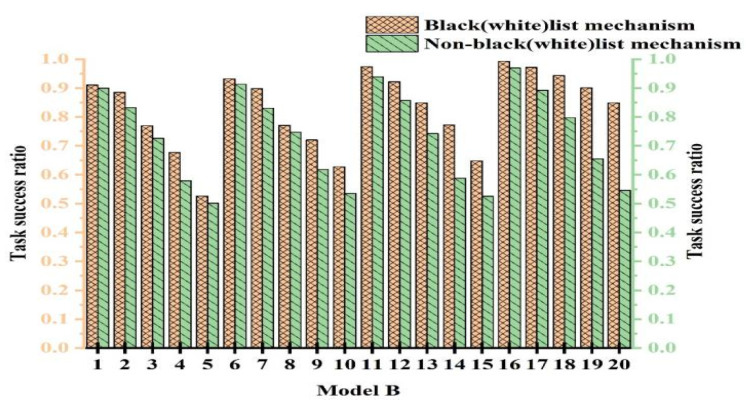
Task success ratio with the black- and whitelist mechanism under Attack Model B.

**Figure 10:**
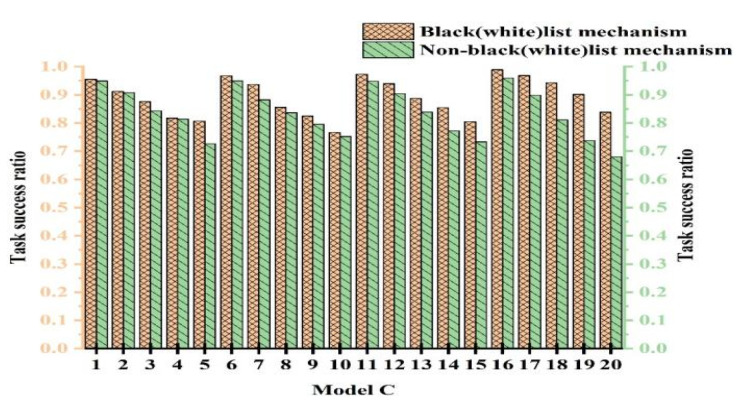
Task success ratio with the black- and whitelist mechanism under Attack Model C.

To validate the efficacy of the black- and whitelist mechanism, we conducted comparative experiments with and without its use. To ensure fairness, multiple experiments were conducted to determine parameter values. The experiments were categorized into 20 cases. The cases from 1 to 5 represent experiments with PCD = 10% and attack model proportions of 10%, 20%, 30%, 40%, and 50%, respectively. The cases from 6 to 10 were with PCD = 20% and attack model proportions of 10%, 20%, 30%, 40%, and 50%, respectively. Figure 8, Figure 9, and Figure 10 show the experimental results for Attack Models A, B, and C, respectively.

From Figure 8, Figure 9 and Figure 10, we observe that the performance with the black- and whitelist mechanism surpassed that without it. Furthermore, as the proportion of attack models increased, the superiority of the mechanism became more pronounced. It can be seen from Figure 8 that only when PCB = 20% and the proportion of attack models was 30%, the same effect was achieved with and without black- and whitelists. However, in other cases, better results were achieved. The combined mechanism improved the task success rate by 1.7~14.6% under 50% attack conditions. The performance margins were limited in low-attack scenarios due to baseline saturation.

This is because when resources were abundant and the proportion of attack models was small, most services using the black- and whitelist mechanism interacted with devices on the whitelist. This is similar to users not employing the mechanism. Consequently, both approaches exhibited a high success rate, higher than the outcomes without any mechanism. However, when resources were limited and the proportion of attack models was large, the black- and whitelist mechanism played a crucial role. Malicious nodes were blacklisted and prohibited from interaction, thereby enhancing the success rate. In contrast, without the black- and whitelist mechanism, the success rate significantly decreased due to extensive attacks. Based on the experimental results, we conclude that the black- and whitelist mechanism was effective in resisting malicious attacks.

### 4.4. Trust Model’s Resistance to Attacks

**Figure 11:**
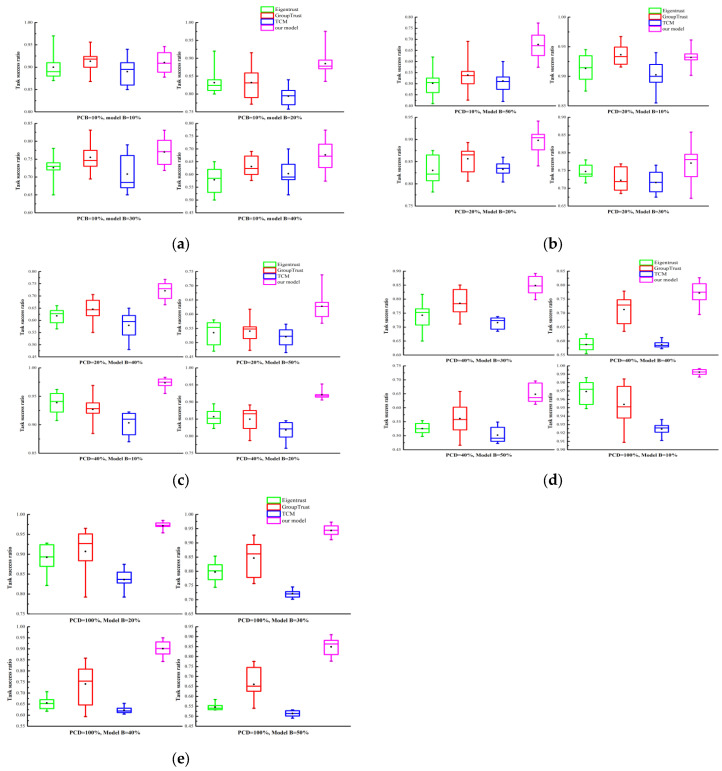
Task success ratio comparison with different trust models: (**a**) PCD = 10%; (**b**) PCD = 10% and PCD = 20%; (**c**) PCD = 20% and PCD = 40%; (**d**) PCD = 40% and PCD = 100%; (**e**) PCD = 100%.

Figure 11 presents the performance comparison of the proposed model with TCM [28], EigenTrust [29], and GroupTrust [30]. EigenTrust and GroupTrust were selected because distributed P2P technology is similar to the edge computing model. GroupTrust extends EigenTrust, and our proposed model further extends the P2P concept to the collaborative edge computing scenario. Therefore, we compared them to determine whether our proposed algorithm outperforms these existing algorithms.

We choose TCM because it is a novel trust mechanism for FIoT systems. It is an adaptive model based on information entropy theory that calculates a device’s global trust value from local evaluation information. In the scenario with PCD = 10% and attack model B, the results are shown in Figure 11a,b. When the proportion of malicious users was 10%, 20%, 30%, and 40%, the proposed model demonstrated the best performance, with task completion rates of 91.026%, 88.501%, 76.990%, and 67.677%, respectively. However, with 50% malicious users, the proposed model performed slightly worse than the GroupTrust model due to randomness from insufficient evaluation information. As the proportion of malicious users increased, the system performance gradually declined. With PCD = 20% and attack model B, the number of user interactions increased significantly. The results are shown in Figure 11b,c. Compared to the other three models, the proposed model achieved the best performance with malicious user proportions of 20%, 30%, 40%, and 50%. However, due to insufficient trust evaluation information, the proposed model could not accurately assess user credibility and achieved a slightly lower task completion rate than the GroupTrust model when malicious users were at 10%. Compared to PCD = 10%, the trust evaluation information was increased at PCD = 20%, leading to some improvement in the system’s task completion rate. With PCD = 40% and attack model B, user interactions were more frequent. The results are shown in Figure 11c,d. With increased trust evaluation information, the proposed model evaluated user credibility more accurately and achieved optimal performance under various malicious user proportions. Compared to PCD = 20%, the system’s task success rate was further improved. With PCD = 100% and attack model B, the edge computing system was extremely busy. The results are shown in Figure 11d,e. Similar to PCD = 40%, the proposed model achieved optimal performance under different malicious user proportions. Compared to PCD = 40%, the system’s task completion rate was significantly improved. Even with 50% malicious users, the proposed model achieved a task completion rate of 84.907%. Figure 10 only shows results for attack model B; similar results were obtained for attack models A and C.

### 4.5. Performance Evaluation of Resource Allocation Algorithms

To demonstrate the performance of the proposed resource allocation algorithm, we verified it through comparative experiments. Since there is no publicly available dataset for end user evaluations of collaborative service provision devices, we conducted our experiments using simulated datasets. The dataset included end user IDs, collaborative service equipment IDs, and service evaluation values from end users to the collaborative service providing equipment. We simulated a total of 18,599 records from 1879 users, with service evaluation values randomly generated in the range of [−2,2]. Based on these evaluations, we formed a collaborative service equipment evaluation network. In this experiment, each algorithm generated a service device allocation list, and the top n devices were recommended to the user. We set n to 5, 10, 15, 20, and 25. We adopted 10-fold cross-validation and used the average as the final result. The evaluation metrics used were precision, recall, and normalized discounted cumulative gain (NDCG).

1. Performance Evaluation of Resource Allocation Algorithms

To validate the performance of the proposed algorithm, we conducted comparative experiments using the BPR, CDAE, and CFGAN algorithms.

BPR [31]: This algorithm employs Bayesian analysis to derive the maximum a posteriori probability estimate. Using stochastic gradient descent, it obtains the parameters of the MF and KNN algorithms, then sorts and assigns them.

CDAE [32]: This algorithm utilizes denoising autoencoders to perform sorting and assignment.

CFGAN [33]: This algorithm adopts a collaborative filtering framework based on generative adversarial networks, effectively a modified version of the GAN algorithm.

To ensure fairness in the comparative experiments, we set the parameters of the aforementioned algorithms according to their respective literature or experimental results.

Under these parameter settings, the comparison algorithms achieved optimal performance. For the BPR algorithm: parameters $\lambda_{u}=\lambda_{i}=0.001$ and $\lambda_{b}=\lambda_{s}=0.2$; for the CDAE algorithm: parameters $\lambda_{u}=\lambda_{i}=0.01$, $\lambda_{b}=\lambda_{s}=0.2$, β=1, and $\eta \in \left\{1,0.1,0.01,0.001\right\}$; for the CFGAN algorithm: parameter $\alpha \in \left\{0.5,0.25,0.1,0.05,0.01\right\}$.

**Figure 12:**
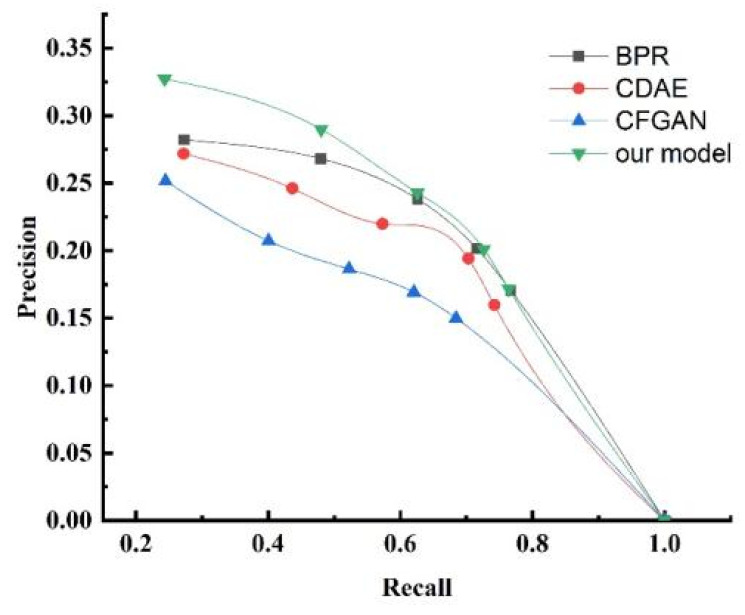
Precision–recall curve.

The experimental results are presented in Table 2, Table 3, Table 4, Table 5 and Table 6. The results reveal that as the number of allocated service devices increased, the precision value decreased accordingly. This indicates that the recommendation accuracy declined to varying degrees, but the decline of the proposed algorithm was less than that of the other algorithms. Conversely, the recall value correspondingly increased. This implies that the recommendation recall rate increased to varying degrees, and the increase of the proposed algorithm was higher than that of the other algorithms. To better illustrate the algorithm’s performance, we plotted the precision–recall curve. Figure 12 shows the precision–recall curve. From the figure, we observe that the proposed algorithm had a larger coverage area, suggesting it is superior to the other three algorithms in balancing precision and recall. In the experimental results, the NDCG index gradually increased. When n=5, compared to the BPR algorithm—which had the highest NDCG among the comparison algorithms—the proposed algorithm showed a 30.68% increase in NDCG. As n increased, the rate of improvement declined, with a 3.34% increase when n=25. This indicates that the recommendation ranking outcomes were continuously improving, and the algorithm presented in this paper outperformed the other algorithms. In conclusion, compared with the BPR, CDAE, and CFGAN algorithms, the proposed approach generated a superior allocation scheme.

2. Impact of Parameters on the Resource Allocation Model

**Figure 13:**
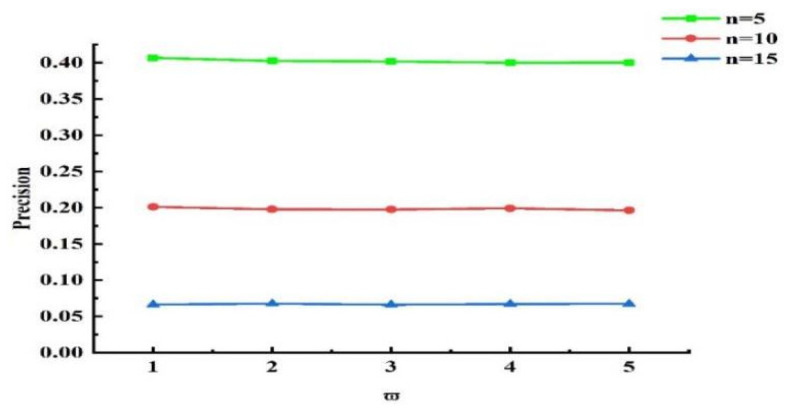
Effect of $\bar{\omega }$ on the Precision index.

**Figure 14:**
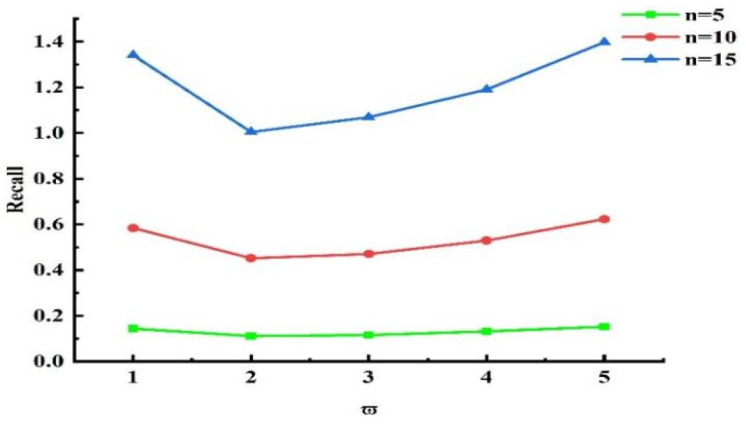
Effect of $\bar{\omega }$ on the Recall index.

**Figure 15:**
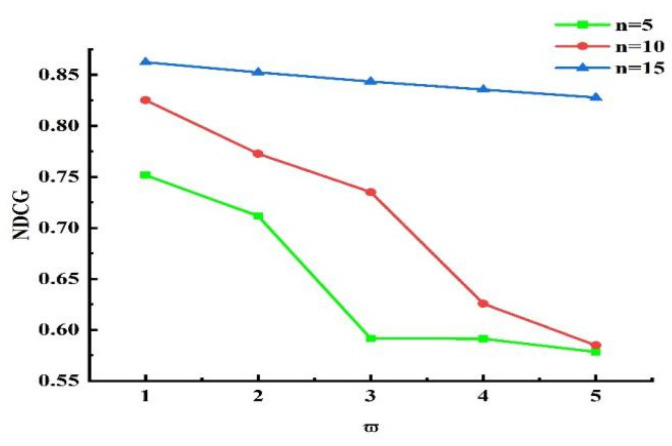
Effect of $\bar{\omega }$ on the NDCG index.

**Figure 16:**
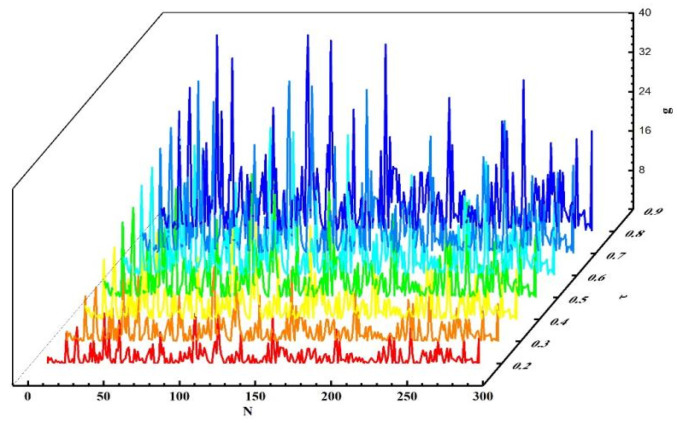
Experimental results with different τ values.

Next, experiments were conducted to validate the influence of the grouping threshold on the algorithm’s outcomes. When τ=0.5 and other values remained unchanged, we obtained different experimental results by varying the value of $\bar{\omega }$. The experimental results for n=5, n=10, and n=15 are presented in Figure 13, Figure 14, and Figure 15, respectively. Analyzing the results in the figures, we observe that under the same n, as $\bar{\omega }$ increased, precision declined, recall rose, and NDCG dropped. This indicates that the smaller $\bar{\omega }$ is, the more data are placed into set ℘. Devices with more effective interactions and higher trust values are more likely to be assigned, resulting in higher precision. As $\bar{\omega }$ increased, the data in set ℘ decreased, and the number of devices directly allocated decreased, leading to decreases in precision and NDCG. Next, experiments were conducted to validate the impact of τ on the grouping outcomes. Keeping the coefficient fixed at 0 and other values unchanged, we obtained different experimental results by varying τ values (0.2, 0.4, 0.6, and 0.8, respectively). The experimental findings are presented in Figure 16. From the figure, we observe that different parameter values had diverse effects on the grouping function value. As the weight of activity degree increased, the grouping function value underwent significant changes. When the grouping value was positive, the grouping function value gradually rose as the parameter value increased. This indicates that services offering more effective interactions and possessing higher trust values were more likely to enter the set ℘ and be allocated. Services that provided less effective interactions and had lower trust values had difficulty entering the direct allocation set.

## 5. Conclusions and Future Works

In this paper, we firstly propose a dynamic trust mechanism that combines subjectivity and objectivity to address two problems in existing trust mechanisms. First, subjective trust mechanisms cannot select a wide range of interaction objects, thereby reducing their performance. Second, objective indirect trust mechanisms ignore the individual perception evaluations of end users. The proposed mechanism incorporates subjective direct trust, objective indirect trust, and an aggregate trust mechanism. Secondly, to address the problem of selecting trusted devices among collaborative service devices, we proposed a selection mechanism based on a collaborative filtering dynamic black- and whitelist. Thirdly, after selecting the trusted devices, we proposed an allocation algorithm based on coalition game theory to allocate the resources of the cooperative service devices. Experimental results demonstrate that the proposed trust mechanism effectively promotes cooperation among multiple devices. It more reliably resists malicious attacks and is suitable for FIoT edge computing systems.

In future work, we plan to conduct targeted experiments involving Sybil attack and Denial-of-Service (DoS) attack models. The Sybil attack, characterized by the creation of multiple fake identities to distort trust evaluation, poses a significant threat to the integrity of trust-based systems. Meanwhile, DoS attacks exploit resource exhaustion vulnerabilities to disrupt system availability. By simulating these attack scenarios, we aim to further evaluate the robustness and resilience of our trust management and resource allocation framework. Moreover, we intend to test the system using real-world datasets or on actual testbeds, such as edge computing platforms or IoT environments, to validate its practical effectiveness. To adaptively respond to dynamic threats and fluctuating environments, we will also integrate an adaptive learning mechanism. This mechanism will dynamically adjust key parameters—such as trust thresholds, activity weights, and coalition grouping factors—based on real-time feedback and historical data. This enhancement is expected to significantly improve the system’s adaptability, stability, and overall decision-making accuracy.

## Figures and Tables

**Table 1:** Simulation experiment settings.

Symbol	Description	Value
N	Quantity of Users	1000
$\delta_{i}$	User Tolerance	10
$\eta_{i}$	User Satisfaction	10
PCD	Percentage of Busy Collaborative End Users	10%, 20%, 40%, 100%
θ	Predefined Number of Trusted Cooperative Service Devices	5
ζ	Total Services	2000
μ	Initial Number of Services per End User	15
$b_{p}$	The Benefits of an Excellent Service	7
$c_{p}$	The Cost of Providing an Excellent Service	−1
$b_{n}$	Loss of Invalid Services	−15
$c_{n}$	The Cost of Ineffective Services Provided	−10

**Table 2:** Comparison results of the algorithms when n=5.

Algorithm	Precision	Recall	NDCG
BPR	0.282097	0.27281	0.509021
CDAE	0.271815	0.271885	0.474660
CFGAN	0.251634	0.244755	0.456654
Our model	0.327279	0.243134	0.565214

**Table 3:** Comparison results of the algorithms when n=10.

Algorithm	Precision	Recall	NDCG
BPR	0.268145	0.479976	0.587745
CDAE	0.246056	0.436588	0.536539
CFGAN	0.207251	0.400631	0.497778
Our model	0.289912	0.480077	0.623811

**Table 4:** Comparison results of the algorithms when n=15.

Algorithm	Precision	Recall	NDCG
BPR	0.238172	0.626019	0.65792
CDAE	0.219814	0.573304	0.600307
CFGAN	0.186295	0.522359	0.556231
Our model	0.242877	0.625874	0.682097

**Table 5:** Comparison results of the algorithms when n=20.

Algorithm	Precision	Recall	NDCG
BPR	0.201455	0.715859	0.696002
CDAE	0.194188	0.702956	0.675496
CFGAN	0.169203	0.620556	0.603054
Our model	0.201123	0.726377	0.737693

**Table 6:** Comparison results of the algorithms when n=25.

Algorithm	Precision	Recall	NDCG
BPR	0.170613	0.766883	0.715204
CDAE	0.159689	0.742395	0.674349
CFGAN	0.149896	0.68435	0.630644
Our model	0.171644	0.764024	0.739104

## Data Availability

All data for this article can be obtained by email from the corresponding author.
